# Programmed death ligand 2 expression plays a limited role in adenocarcinomas of the gastroesophageal junction after preoperative chemotherapy

**DOI:** 10.1007/s10353-021-00700-4

**Published:** 2021-05-05

**Authors:** Gerd Jomrich, Dagmar Kollmann, Lavinia Wilfing, Sanja Radosavljevic, Dariga Ramazanova, Robin Ristl, Richard P. Grose, Aysegül Ilhan-Mutlu, Matthias Preusser, Christina Fassnacht, Yi-Chien Tsai, Emmanuella Guenova, Sebastian F. Schoppmann

**Affiliations:** 1 Department of Surgery, Comprehensive Cancer Center Vienna, Upper-GI-Service, GET-Unit, Medical University of Vienna, Spitalgasse 23, 1090 Vienna, Austria; 2 Section for Medical Statistics (IMS), Center of Medical Statistics, Informatics and Intelligent Systems, Medical University of Vienna, Spitalgasse 23, 1090 Vienna, Austria; 3 Centre for Tumour Biology, Barts Cancer Institute, Queen Mary University of London, John Vane Science Centre, Charterhouse Square, EC1M 6BQ London, UK; 4 Division of Oncology, Department of Medicine I and Comprehensive Cancer Center, GET-Unit, Medical University of Vienna, Spitalgasse 23, 1090 Vienna, Austria; 5 Department of Dermatology, University Hospital Zurich and Faculty of Medicine, University of Zurich, Raemistrasse 100, 8091 Zurich, Switzerland; 6 Department of Dermatology, Lausanne University Hospital (CHUV) and Faculty of Biology and Medicine, University of Lausanne, Av de Beaumont 29, 1011 Lausanne, Switzerland

**Keywords:** PD-L2, Adenocarcinoma of the gastroesophageal junction, Neoadjuvant therapy, Immunotherapy

## Abstract

**Background:**

The effects of cytotoxic chemotherapy on the expression of programmed death ligand 2 (PD-L2) are unknown and little is known about how the tumor microenvironment changes following neoadjuvant chemotherapy in locally advanced gastroesophageal adenocarcinomas (AEG). Recently, a number of studies reported that cytotoxic chemotherapy affects the expression levels of programmed cell death protein 1 (PD-1) and its ligand 1 (PD-L1). Regarding PD-L2, the second known ligand of PD‑1, no data on potential changes in expression patterns in patients with preoperatively treated AEG are available. The aim of this study was to investigate the impact of cytotoxic chemotherapy on PD-L2 expression in patients with resectable AEG.

**Methods:**

Consecutive patients with locally advanced AEG treated with preoperative cytotoxic chemotherapy were included. PD-L2 expression by cancer cells (CCs) and tumor-infiltrating lymphocytes (TILs) was investigated in samples of paired diagnostic biopsies and resected tumor specimens by immunohistochemistry using two different anti-PD-L2 antibodies.

**Results:**

Included were 40 patients with AEG and available paired tumor tissue samples. PD-L2 expression was observed in one diagnostic biopsy sample by CCs and in one diagnostic biopsy sample by TILs. There was no difference concerning the expression levels measured by the two antibodies.

**Conclusion:**

In contrast to previously published studies reporting PD-L2 expression rates of up to 50% in AEGs, in our cohort, PD-L2 expression seems to play no significant role in AEG.

## Introduction

Gastroesophageal adenocarcinoma (AEG) is one of the ten most common causes of cancer deaths worldwide [1, 2]. Even though the use of multimodal therapies combining surgery, cytotoxic chemotherapy, radiotherapy, and targeted therapy has improved patientsʼ survival rates, 5‑year survival rates remain poor, at 10–15% [3–7]. The majority of patients present with locally advanced AEG and are therefore eligible for preoperative treatment [8, 9]. Perioperative chemo(radio)therapy along with surgery is now the standard of care treatment for patients with locally advanced AEG [10, 11]. However, due to the development of resistance to cytotoxic chemotherapy, these conventional therapies are limited in efficacy. Recently, targeted therapy strategies have become a promising approach to overcome resistance to cytotoxic chemotherapies. Human epidermal growth factor receptor 2 (HER-2) has been confirmed to play an important role in the treatment of AEG. Cytotoxic chemotherapy in combination with trastuzumab, a monoclonal antibody against HER‑2, significantly improves the prognosis in patients with HER-2-positive AEG [12]. Despite these promising data, recent studies also point out emerging resistance mechanisms to anti-HER2 therapy [13]. Therefore, new approaches for molecular-targeted therapeutics are needed to further improve patients’ survival. Cancer immunotherapy has revolutionized the field of oncology and immune checkpoint therapy provides promising treatment results in various cancer entities, including esophageal cancer [14, 15]. The therapeutic modulation of the PD‑1 pathway has emerged as a promising target in the treatment of melanoma, renal cancer, non-small cell lung cancer, and urothelial cancer [16]. Programmed cell death 1 (PD-1) is one of the most potent immune-checkpoint molecules, expressed mainly on the surface of T‑cells during activation. Binding of this co-receptor results in limited activation and function of the T‑cell and is therefore of great importance in avoiding hyperactivation [17]. Cancer cells can take advantage of this mechanism and misuse it to hide from immunosurveillance [18]. Programmed death ligand 1 (PD-L1) and programmed death ligand 2 (PD-L2) are cell surface glycoproteins belonging to the B7 family and are the two known ligands for PD‑1 [19, 20].

In advanced esophageal squamous cell cancer (ESCC), pembrolizumab, a monoclonal antibody targeting PD-L1, has been approved by the United States Food and Drug Administration (FDA) and significantly improves patient survival rates [21]. Recently, several studies could show that cytotoxic chemotherapy can affect the expression of PD-L1. So far, our group has investigated the prognostic value of PD‑1, PD-L1, and PD-L2 regarding survival rates of patients with AEG [22, 23]. Since neoadjuvant chemotherapy has become indispensable in the treatment of patients with locally advanced AEG, it is necessary to understand its impact on immune characteristics. To the best of our knowledge, there are no data on the neoadjuvant chemotherapeutic effect on the expression of PD-L2 in AEG until today. Therefore, the aim of this study was to investigate the potential change in the expression level of PD-L2 in neoadjuvantly treated AEG patients.

## Materials and methods

### Patients

Patients with histologically confirmed and locally advanced AEG who underwent neoadjuvant chemotherapy (NCT) followed by curative resection between 2003 and 2016 at the Department of Surgery of the Medical University of Vienna were enrolled in this study. Patients’ demographic and histopathological characteristics were collected from a prospectively maintained database. The pathological tumor–node–metastasis (TNM) classification of the Union for International Cancer Control (UICC), 8th edition, was used to determine the clinical tumor stage pre-NCT and post-surgery [24]. Patients with complete response to the neoadjuvant chemotherapy, distant metastasis at the time of surgery, positive resection margins, or malignancies other than AEG were excluded. All patients were discussed in multidisciplinary tumor board meetings and either received an oxaliplatin/capecitabine-based or a cisplatin/5-fluoruacil-based neoadjuvant chemotherapy according to the standards of the Comprehensive Cancer Center of the Medical University of Vienna. Depending on the primary tumor localization, patients either underwent abdominothoracic en-bloc esophagectomy or trans-hiatal extended gastrectomy.

Paired diagnostic biopsies and surgical specimens were acquired from each patient included in the study. Biopsy specimen were obtained during esophagogastroduodenoscopy (EGD) according to the standards of the European Society of Gastrointestinal Endoscopy (ESGE) [25, 26]. PD-L2 expression was analyzed by two different immunohistochemical staining methods. This study was approved by the Ethics Committee of the Medical University of Vienna, Austria, and was performed in accordance with the Declaration of Helsinki (EK 1056/2016).

### Assessment of response to NCT

The response to neoadjuvant chemotherapy was analyzed through histopathological investigation of the resection specimens according to the Mandard grading system [27]. In brief, this classification divides the histopathological effects into five tumor regression grades (TRGs) based on the ratio of vital tumor tissue and fibrosis: TRG 1—complete regression (=fibrosis without detectable tumor tissue); TRG 2—fibrosis with scattered tumor cells; TRG 3—fibrosis and tumor cells with predominance of fibrosis; TRG 4—fibrosis and tumor cells with predominance of tumor cells; TRG 5—tumor tissue without changes of regression [27].

### Immunohistochemistry

Paraffin-embedded specimens fixed in 4% buffered formalin were cut into 4‑µm-thick slides and subjected to immunohistochemical analyses. The following antibodies were utilized for PD-L2 detection: rabbit anti-human PD-L2 antibody (Cell Signaling Technology, Cambridge, United Kingdom, #82723, clone D7U8C, dilution 1:50) and the anti-PD-L2 antibody (Proteintech, Manchester, United Kingdom, #18251-1-AP, dilution: 1:200). The sections were deparaffinized and rehydrated in graded series: X‑TRA-Solv 8 (Medite, Burgdorf, Germany, # 41-5212-00) for 15 min at 68 °C; xylol—5 min RT, 100% EtOH—5 min RT; 96% EtOH—5 min RT; 80% EtOH—5 min RT; distilled water 2 min RT. The antigens were retrieved via cooking at 100 ºC for 45 min with Leica, Vienna, Austria, buffer nr. 2. Goat anti-rabbit IgG (H+L), HRP-conjugated antibody (Abcam, Cambridge, United Kingdom, #ab97051) was used as a secondary antibody. The stainings with the primary and secondary antibodies were performed according to the manufacturer’s protocols. In order to depict the cell nuclei, sections were counterstained with Mayer’s hematoxylin.

For each patient, histologic expression of PD-L2 was analyzed separately for tumor cells and TILs. The percentage of tumor cells and lymphocytes showing immunoreactivity to PD-L2 was rated (positive staining, 0–100%) and classified as follows: 0: no positive cells; +: 5–25% of cells; ++: 26–50% of cells; +++: 51–75% of cells; and ++++: 76–100% of cells. Histological analyses were performed by two pathologists who were blinded to the clinical characteristics of each patient.

### Statistical analysis

Statistical analysis was performed using the R 4.0.3 (R Foundation for Statistical Computing, Vienna, Austria, www.r-project.org). Correlations between clinicopathological factors and expression of PD-L2 were analyzed with the Kendall’s correlation coefficient (tau-b).

## Results

A total of 40 paired sets of diagnostic biopsies and surgical specimens from patients with neoadjuvantly treated AEG were included in this study. The median age of the patients in the study population was 64 years (range: 35.0–80.5 years) and the ratio of female to male was 1:2.3. The most frequent tumor localization (28 patients, 70.0%) was AEG type I according to the Siewert classification [28]. Before neoadjuvant chemotherapy, the majority of patients were diagnosed with UICC stage III (30 patients, 75.0%). After surgical resection, UICC stage III (25 patients, 62.5%) remained the most frequent staging. 23 (57.5%) patients presented with a moderately differentiated tumor (G2). Regarding the response to administered chemotherapy, 18 patients (45.0%) showed partial response (TRG 2–3), 15 patients (37.5%) showed minor regression (TRG 4), and 7 patients (17.5%) had no signs of regression at all (TRG 5). 34 (85.0%) patients presented with an ECOG (Eastern Cooperative Oncology Group) score of 0 before neoadjuvant chemotherapy and 15 patients (37.5%) were classified as ASA (American Society of Anesthesiologists) 1 before surgery. Clinico-pathological data are presented in Tab. 1.

### PD-L2—cancer cells (CC) and tumor-infiltrating lymphocytes (TILs)

**Fig. 1:**
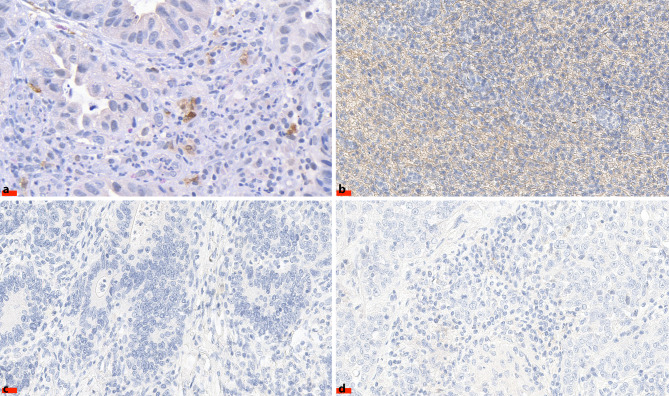
Representative images of diagnostic biopsies (**a,** **b**) and surgical specimen (**c,** **d**) of adenocarcinomas of the gastroesophageal junction stained for programmed death ligand 2 (PD-L2). **a** Positive signals of PD-L2 expression in cancer cells and **b** in tumor-infiltrating lymphocytes. **c** No signal or low expression of PD-L2 in cancer cells and **d** in tumor-infiltrating lymphocytes. Scale bar = 20 µm. Original magnification ×400 all

A total of 33 cases were evaluated regarding the change of PD-L2 expression by CC and TILs in diagnostic biopsies. On 7 specimens of diagnostic biopsies, IHC could not be performed due to technical problems. All of the 40 surgical specimens were successfully stained. Among the specimens of diagnostic biopsies, one sample showed 1+ (1–25%) PD-L2 staining by cancer cells and another sample 2+ (26–50%) PD-L2 staining by TILs (same results for both PD-L2 antibodies; Fig. 1a, b). Both cases showed Mandard TRG 2. None of the surgical specimens showed positive staining for PD-L2 with either of the two used antibodies (Fig. 1c, d). Based on these results, no further meaningful investigations of the changes of PD-L2 expression have been conducted (Tab. 2).

## Discussion

The incidence of adenocarcinoma of the esophagogastric junction (AEG) has increased markedly over the past years in the western world. Its aggressive nature leads to early local invasion and systemic spreading [1, 2]. In recent years, cancer immunotherapy has become another pillar in cancer therapy. The clinical use of monoclonal antibodies targeting the PD‑1 pathway has achieved major improvements in patient survival in multiple malignancies. The immune suppressive proteins PD-L1 and PD-L2 limit T‑cell activation and cytokine production, which contributes to immunosurveillance [14, 29–32]. Compared to PD-L1, the relevance of PD-L2 tumor-expression has not been explored intensively. Regarding AEG, only little information is available on its immune characteristics. Derks et al. were able to detect PD-L2 expression by cancer cells in 52% of patients with esophageal adenocarcinoma [33]. Recently, our group investigated the role of PD1, PD-L1, and PD-L2 expression as a prognostic factor in patients with resectable AEG. In that study, PD-L2 expression was only occasionally found on TILs and cancer cells (1.8 and 3.5%, respectively) [22, 23].

The standard approach for the treatment of patients with locally advanced AEG is cytotoxic chemotherapy or chemoradiotherapy followed by surgery. The use of cytotoxic chemotherapy is inevitable for treating advanced cancers. These drugs target nucleic acid metabolism or interfere with the microtubule network of proliferating tumor cells. In recent years, various studies reported that PD-L1 expression can be either decreased or increased by neoadjuvant chemotherapy according to the cancer type. These results suggested that the immune response, altered by the chemotherapeutical drugs, could be inhibited by upregulated PD-L1, which may play a role in chemoresistance [30, 31, 34–40]. The potential change in PD-L2 expression has hardly been researched; regarding AEG, no data were available until now. With chemoresistance being a growing problem, it is important to further investigate the effects of chemotherapy on PD-L2 expression to gain more understanding of this issue in anti-cancer therapy.

In this study, we aimed to investigate the potential effect of oxaliplatin/capecitabine- and cisplatin/5-fluorouracil-based neoadjuvant therapy on the expression of PD-L2 in cancer cells and TILs in neoadjuvantly treated AEG using immunohistochemistry.

In our study, PD-L2 expression by AEG cancer cells and TILs was detected in only one out of 33 specimens of diagnostic biopsies. All the 40 surgical (post neoadjuvant therapy) specimens were rated as negative regarding PD-L2 expression. Therefore, we were not able to further investigate/illustrate a potential correlation between neoadjuvant treatment and the expression pattern of PD-L2. The positive expression by 6% of the specimens of diagnostic biopsies is a result that needs to be validated in a larger study population.

We have to consider certain limitations of our study. There is a potential selection bias, caused by only partial availability of tumor tissue, especially of diagnostic biopsies. The small amount of tissue obtained from a biopsy might not be sufficient to represent the PD-L2 expression rate of the tumor. Further, the retrospective nature of this single-center study represents another limitation. In general, the immunohistochemical methods for PD-L2 are not yet well established compared to those for PD-L1. Dhupar et al. have proposed that different criteria for positive staining of PD-L2 are a major reason for the generally inconsistent results [41]. Another aspect would be that patient cohorts from different studies vary regarding their demographic and pathologic factors, which can also affect the results of PD‑L expression analysis. Regarding the evaluation of the immunohistochemical expression grade, there is always a chance of interobserver variability, especially in borderline cases. Last but not least, an important limitation to mention, is the small study population.

Surprisingly, we found no PD-L2 expression in postsurgical specimens in this study. These findings are in contrast with our previously published data on the expression of PD-L2 as a potentially prognostic factor in AEG, describing 3.5% positive PD-L2 cases in patients with primarily resected and neoadjuvantly treated AEG [22]. The different expression rate may result from the inconsistent study populations, but it could also be interpreted as a small indication for a negative effect of the chemotherapy on PD-L2 expression. On the other hand, the non-existent PD-L2 expression in the present study is possibly related to the used antibodies. The issue of controversies attributed to different antibody specificity that leads to both underestimating and overestimating the results has been reported in various studies investigating PD-L1 and PD-L2 expression [41–43].

To the best of our knowledge, this is the first study investigating the expression of PD-L2 in neoadjuvantly treated AEG patients.

In conclusion, we were not able to demonstrate a significant change in the expression of PD-L2 in AEG patients given neoadjuvant treatment. Our results can be interpreted as a tendency and definitely need further investigation. It is of great importance to gain better understanding into how cytotoxic chemotherapy affects PD-L2 expression and function to potentially contribute to develop new and highly effective anti-cancer therapies for patients suffering from AEG.

**Table 1:** Clinicopathologic parameters of adenocarcinomas of the gastroesophageal junction

	All patients	
Factors	(*n* = 40)	(%)
*Age, mean (years)*	64 (35.0–80.4)	–
*Sex*		
Male	30	(75.0)
Female	10	(25.0)
*Tumor differentiation*		
1	1	(2.5)
2	23	(57.5)
3	16	(40.0)
*cT before NCHT*		
2	17	(42.5)
3	22	(55.0)
4	1	(2.5)
*cN before NCHT*		
0	5	(12.5)
1	29	(72.5)
2	6	(15.0)
*pT*		
1	8	(20.0)
2	11	(27.5)
3	19	(47.5)
4	2	(5.0)
*pN*		
0	16	(40.0)
1	12	(30.0)
2	12	(30.0)
*Mandard TRG*		
2	8	(20.0)
3	10	(25.0)
4	15	(37.5)
5	7	(17.5)
*Siewert classification*		
AEG I	28	(70.0)
AEG II	6	(15.0)
AEG III	6	(15.0)
*NCHT regime*		
Cisplatin/5-fluoruacil	21	(52.5)
Oxaliplatin/capecitabine	19	(47.5)
*Adjuvant chemotherapy*		
No	29	(72.5)
Yes	11	(27.5)
*Surgical approach*		
Abdominal	12	(30.0)
Thoracoabdominal	28	(70.0)
*ASA*		
1	15	(37.5)
2	21	(52.5)
3	4	(10.0)
*ECOG before NCHT*		
0	34	(85.0)
1	5	(12.5)
2	1	(2.5)
*ECOG before resection*		
0	30	(75.0)
1	8	(20.0)
2	2	(5.0)

*cT* clinical tumor stage, *cN* clinical lymph node stage, *pT* pathological tumor stage, *pN* pathological lymph node stage, *NCHT* neoadjuvant chemotherapy, *AEG* adenocarcinoma of the gastroesophageal junction, *ASA* American Society of Anesthesiologists, *ECOG* Eastern Cooperative Oncology Group

**Table 2:** Expression of PD-L2 before and after neoadjuvant chemotherapy

	Antibody		Antibody	
	#82723		#18251-1-AP	
**Specimen of diagnostic biopsies before NCHT**	(*n* = 33)	(%)	(*n* = 33)	(%)
*PD-L2 expression in cancer cells*				
0 (0–4%)	32	(96.9)	32	(96.9)
1+ (5–25%)	1	(3.1)	1	(3.1)
2+ (26–50%)	0	0	0	0
3+ (51–75%)	0	0	0	0
4+ (76–100%)	0	0	0	0
*PD-L2 expression in TILS*				
0 (0–4%)	32	(96.9)	32	(96.9)
1+ (5–25%)	1	(3.1)	1	(3.1)
2+ (26–50%)	0	0	0	0
3+ (51–75%)	0	0	0	0
4+ (76–100%)	0	0	0	0
**Specimen of surgical resection after NCHT**	(*n* = 40)	(%)	(*n* = 40)	(%)
*PD-L2 expression in cancer cells*				
0 (0–4%)	40	(100)	40	(100)
1+ (5–25%)	0	0	0	0
2+ (26–50%)	0	0	0	0
3+ (51–75%)	0	0	0	0
4+ (76–100%)	0	0	0	0
*PD-L2 expression in TILs*				
0 (0–4%)	40	(100)	40	(100)
1+ (5–25%)	0	0	0	0
2+ (26–50%)	0	0	0	0
3+ (51–75%)	0	0	0	0
4+ (76–100%)	0	0	0	0

*NCHT* neoadjuvant chemotherapy, *TILs* tumor infiltrating lymphocytes, *PD-L2* programmed death ligand 2
